# HDAC4 Promotes Cancer Stemness in Nonsmall Cell Lung Cancer by Upregulating SLC38A2 Expression via HIF‐1α to Enhance Glutamine Metabolism

**DOI:** 10.1155/ancp/2525721

**Published:** 2026-09-28

**Authors:** Changxian Chen, Xiaoming Jiang, Zhenwu Yao, Jiaxue Sun, Liju Zhang, Mingyu Xu, Weimin Bao, Weijun Liu

**Affiliations:** ^1^ Department of Anorectal Diseases, The First People’s Hospital of Yunnan Province, Kunming University of Science and Technology Affiliated Hospital, Kunming 650032, China, ypfph.com; ^2^ Department of Anorectal Diseases, The First People’s Hospital of Yunnan Province, School of Medicine, Kunming University of Science and Technology, Kunming 650032, China, kmust.edu.cn; ^3^ School of Medicine, Yunnan University, Kunming 650500, China, ynu.edu.cn

**Keywords:** cancer stemness, glutamine metabolism, HDAC4, HIF-1α, NSCLC, SLC38A2

## Abstract

**Background and Purpose:**

The malignant progression of nonsmall cell lung cancer (NSCLC) is closely related to cancer stemness. Histone deacetylase 4 (HDAC4) plays a regulatory role in lung cancer, but its effect on NSCLC stemness remains unclear. This study aimed to investigate the role and mechanism of HDAC4 in NSCLC stemness.

**Methods:**

In this study, a tumor‐bearing model was established by subcutaneously injecting A549 cells into the right dorsal side of nude mice. Reverse transcription quantitative polymerase chain reaction (RT‐qPCR), Western blotting, and immunohistochemistry were used to measure gene and protein expression levels. Flow cytometry, sphere formation assays, and Transwell experiments were employed to assess cancer cell stemness, migration, and invasion. Additionally, a kit was used to measure changes in glutamine metabolism‐related indicators.

**Results:**

In this study, we found that knocking down HDAC4 expression inhibited the expression of SRY‐box transcription factor 2 (SOX2), octamer‐binding transcription factor 4 (OCT4), and nanog homeobox (NANOG) in A549 cells; it also reduced the proportions of CD133‐ and CD44‐positive cells and suppressed their sphere formation, cell migration, and invasion abilities. Furthermore, HDAC4 knockdown inhibited glutamine uptake, glutamate production, α‐ketoglutarate levels, and glutaminase (GLS) activity. Notably, treatment with the additional glutamine metabolism inhibitor CB‐839 attenuated the promoting effects of HDAC4 overexpression on the stemness, migration, and invasion of A549 cells. In addition, HDAC4 promoted the expression of hypoxia‐inducible factor‐1 alpha (HIF‐1α) and solute carrier family 38 member 2 (SLC38A2); after overexpressing HIF‐1α, the inhibitory effect of HDAC4 knockdown on SLC38A2 expression in A549 cells was weakened.

**Conclusion:**

Our study reveals a critical mechanism by which HDAC4 enhances glutamine metabolism through upregulation of HIF‐1α expression to promote SLC38A2 expression, thereby driving NSCLC stemness and progression.

## 1. Introduction

Nonsmall cell lung cancer (NSCLC) is one of the most common malignant diseases worldwide and is a leading cause of cancer‐related death, accounting for ~85% of all lung cancers and posing a serious threat to human health [1]. Although significant progress has been made in recent years in cancer‐related treatments such as chemotherapy, radiotherapy, and immunotherapy, the 5‐year overall survival rate for patients with NSCLC remains low at ~15% [2]. Notably, the poor prognosis of NSCLC is closely associated with the acquisition of cancer cell stemness [3]. Cancer cell stemness not only endows tumor cells with self‐renewal capabilities to promote tumorigenesis but also leads to treatment resistance, tumor recurrence, and metastasis [4]. Conversely, inhibiting cancer cell stemness can suppress the malignant progression of NSCLC [5]. Therefore, in‐depth research into the regulatory mechanisms of cancer stemness in NSCLC is highly important for its treatment.

Glutamine is a nonessential amino acid that is abundant in the blood, serves as a crucial source of carbon and nitrogen, and plays a significant role in regulating cell proliferation, survival, and function [6]. The process by which glutamine is converted into α‐ketoglutarate to indirectly supply substrates for the citric acid cycle is termed glutamine metabolism [7]. Glutamine metabolism plays a vital role in the growth and proliferation of tumor cells. For instance, triple‐negative breast cancer (TNBC) and KRAS‐mutant colorectal cancer are significantly dependent on glutamine, and survival rates are positively correlated with glutamine metabolism [8, 9]. Furthermore, existing evidence suggests that disrupting glutamine metabolism can inhibit tumor cell self‐renewal capacity by increasing intracellular reactive oxygen species (ROS) levels and downregulating the expression of tumor stemness‐related genes and pluripotency factors [10, 11]. Solute carrier family 38 member 2 (SLC38A2), a glutamine transporter, plays a critical role in glutamine metabolism [12]. SLC38A2 can regulate NSCLC immune responses and subsequent cancer progression by promoting glutamine metabolism [13]. Although the connections among glutamine metabolism, cancer cell stemness, and NSCLC progression have received attention, the intrinsic associations and fine molecular regulatory mechanisms among these three processes have not yet been systematically elucidated. The key regulatory links through which glutamine metabolism and tumor stemness are coupled to each other to jointly drive NSCLC progression remain to be explored.

Histone deacetylase 4 (HDAC4), a member of the class IIa HDAC family, plays vital roles in chromatin remodeling and gene expression regulation [14]. Multiple studies suggest that HDAC4 plays a clear protumorigenic role in cancer: its overexpression drives proliferation, invasion, and metastasis in various tumors, including glioma [15], esophageal cancer [16], and gastric cancer [17]. HDAC4 also contributes to lung cancer progression by regulating epithelial‒mesenchymal transition (EMT), autophagy, and apoptosis [18, 19]. Notably, HDAC4 has been verified to facilitate cancer stemness in glioma [20]. In this study, bioinformatics analysis was performed based on data from The Cancer Genome Atlas (TCGA) and Gene Expression Omnibus (GEO) databases. The results revealed that the expression level of HDAC4 was significantly positively correlated with those of multiple cancer stemness‐related genes (such as SRY‐box transcription factor 2 [SOX2], nanog homeobox [NANOG], CD44, and ALDH1A1) and amino acid metabolism‐related transporters (such as SLC38A2 and SLC7A5). These bioinformatics results suggest that HDAC4 may participate in the progression of NSCLC by modulating cancer stemness and glutamine metabolism. Based on the above evidence, we selected HDAC4 as the core molecule to further explore its roles and underlying mechanisms in the regulation of cancer stemness and glutamine metabolism in NSCLC.

Hypoxia‐inducible factor‐1 alpha (HIF‐1α) is a core transcription factor that regulates tumor metabolic reprogramming and stemness properties and plays a critical role in the malignant progression of NSCLC [21, 22]. Previous studies have confirmed that activation of the HIF‐1α signaling pathway upregulates the expression of key transporters and metabolic enzymes involved in glutamine metabolism, drives cellular glutamine metabolic reprogramming, and subsequently mediates malignant EMT in bronchial epithelial cells [23]. Furthermore, research has revealed that HDAC4 specifically removes acetylation modifications at lysine residues in the N‐terminus of the HIF‐1α protein, stabilizes the HIF‐1α protein, and enhances its transcriptional function, thereby regulating tumor glycolytic metabolism and chemotherapy resistance under hypoxic conditions [24]. Additional studies have demonstrated that in breast cancer, HIF‐1α predominantly regulates the transcriptional activation of the key glutamine transporter SLC38A2 (SNAT2) under hypoxic conditions, mediating abnormally enhanced tumor glutamine uptake [25]. However, although the above studies have revealed the regulatory role of HDAC4 in HIF‐1α protein stability and the transcriptional activation of the glutamine transporter SLC38A2 by HIF‐1α, whether HDAC4 specifically remodels the glutamine metabolic network through fine regulation of the HIF‐1α/SLC38A2 signaling axis and thereby confers cancer cell stemness properties in NSCLC remains systematically unexplored. The cascade regulatory mechanism of the HDAC4–HIF‐1α–SLC38A2 signaling axis in NSCLC proposed in this study complements the multidimensional regulatory network of epigenetic–metabolism–stemness in NSCLC and has clear innovative and specific research value.

Therefore, the aim of the present study was to investigate in depth the specific mechanism through which HDAC4 regulates NSCLC stemness, verify whether it upregulates SLC38A2 expression by stabilizing HIF‐1α, enhances glutamine metabolic flux, and ultimately promotes stemness maintenance and malignant progression in NSCLC, thereby providing a new therapeutic target for clinically blocking tumor metabolic dependence.

## 2. Materials and Methods

### 2.1. Cell Culture and Treatment

In this study, A549 human NSCLC cells were selected for in vitro experiments. This cell line is a classic research model in the field of lung cancer. Moreover, its cell subsets highly express the cancer stem cell markers CD133 and ABCG2 and exhibit typical cancer stemness characteristics, which aligns with the research focus of this study on lung cancer stem cells and glutamine metabolism. A549 cells (SCSP‐503; RRID: CVCL_0023) were obtained from the Cell Bank of the Chinese Academy of Sciences. The cells were cultured in DMEM (11965118, Gibco, USA) supplemented with 10% FBS (A5256701, Gibco, USA), 100 U/mL penicillin, and 100 µg/mL streptomycin (15140122, Gibco, USA).

For lentiviral transfection, A549 cells were seeded into 6‐well plates and cultured until the cell density reached 60%–70%. The cells were then infected with lentiviral vectors at a multiplicity of infection (MOI) of 50. All lentiviral constructs, including sh‐NC, sh‐HDAC4, sh‐HIF‐1α, OE‐NC, OE‐HDAC4, and OE‐HIF‐1α, were designed and packaged by GeneChem (Shanghai, China). The knockdown vectors (sh‐HDAC4, sh‐HIF‐1α, and sh‐NC) use pLKO.1‐puro as the plasmid backbone, while the overexpression vectors (OE‐HDAC4, OE‐HIF‐1α, and OE‐NC) use pLVX‐puro as the plasmid backbone. Lentiviral particles were packaged and produced in 293T cells using the Genma Gene third‐generation four‐plasmid lentiviral packaging system, in which the corresponding transfer vector was cotransfected with the three helper packaging plasmids pGag/Pol, pRev, and pVSV‐G into 293T cells. After 72 h of continuous infection, stably transfected cell lines were screened with 2 μg/mL puromycin (ST551, Beyotime, China). Stable cell phenotypes were verified by Western blotting and reverse transcription quantitative polymerase chain reaction (RT‐qPCR) to confirm successful knockdown or overexpression efficiency. Additionally, to verify whether glutamine metabolism affects the regulatory role of HDAC4 in A549 cell stemness, stably transfected OE‐HDAC4 cells were treated with 2 μM of the glutamine metabolism inhibitor CB‐839 for 24 h for subsequent functional validation.

### 2.2. CCK‐8 Cell Viability Assay

The viability of A549 cells under different treatments was determined using a CCK‐8 assay (C0038, Beyotime, China). Briefly, logarithmic‐phase A549 cells were seeded into 96‐well plates at an appropriate density and cultured overnight for adherence. After the corresponding experimental treatments were performed, 10 μL of the CCK‐8 reagent was added to each well, and the cells were incubated at 37°C for 2 h in the dark. The absorbance value of each well was measured at a wavelength of 450 nm using a microplate reader. Cell viability was calculated according to the measured absorbance values, with blank and control wells used for background correction.

### 2.3. Sphere Formation Assay

A549 cells were treated differently and seeded in ultralow‐adhesion 96‐well plates. They were cultured in serum‐free DMEM/F12 medium (11320033, Gibco, USA) supplemented with 10 ng/mL epidermal growth factor (EGF, E9644, Sigma‒Aldrich, USA), 10 ng/mL basic fibroblast growth factor (bFGF, F0291, Sigma‒Aldrich, USA), 50 ng/mL heparin (HY‐17567, MCE, USA), and 4 ng/mL insulin (Y0001717, Sigma‒Aldrich, USA). The medium was replaced every 3 days. After 14 days of incubation, cell sphere formation was observed under an inverted microscope.

### 2.4. Transwell Assays

Transwell assays were performed to evaluate the migration and invasion abilities of A549 cells after different treatments. For the migration assay, pretreated A549 cells were resuspended in a serum‐free medium and seeded into the upper Transwell chamber. The lower chamber was filled with 500 μL of medium containing 10% FBS to form a chemotactic gradient. For the invasion assay, the upper chamber was precoated with the Matrigel matrix to simulate the extracellular microenvironment, and the subsequent cell seeding and culture conditions were the same as those used for the migration assay. After 24 h of incubation at 37°C, the nonmigrated and noninvaded cells in the upper chamber were gently removed. The cells that migrated or invaded to the lower membrane surface were fixed with 4% paraformaldehyde and stained with crystal violet. Finally, the stained cells were photographed and counted under an inverted microscope for statistical analysis.

### 2.5. RT‐qPCR

Total RNA was extracted from A549 cells treated with different conditions using the TRIzol reagent (R0016, Beyotime, China). The extracted RNA was reverse‐transcribed into cDNA using a reverse transcription kit (T2240, Solarbio, China). qPCR amplification was subsequently performed using cDNA as the template following the instructions provided for the SYBR Green PCR Mastermix (SR1110, Solarbio, China), with β‐actin used as the internal reference. The results were calculated using the 2^−ΔΔCt^ method. The primer sequences are listed in Table 1.

**Table 1:** Primer sequences.

Target	Sequence (F: Forward primer; R: Reverse primer)
HDAC4	F: 5′‐CATCCTTGCCCAACATCAC ‐3′
	R: 5′‐CTCAGGTAGGGAGTGAGGT ‐3′
SOX2	F: 5′‐CGGATTATAAATACCGGCCC ‐3′
	R: 5′‐GTGTACTTATCCTTCTTCATGAGC ‐3′
OCT4	F: 5′‐CCTTCGCAAGCCCTCATTTC ‐3′
	R: 5′‐TAGCCAGGTCCGAGGATCAA ‐3′
NANOG	F: 5′‐GTCTCGTATTTGCTGCATCGT ‐3′
	R: 5′‐ TTCCTTCTCCACCCCAACCA ‐3′
HIF‐1α	F: 5′‐TACCCTCTGATTTAGCATGTAGAC ‐3′
	R: 5′‐TCACAATCATAACTGGTCAGCT ‐3′
β‐actin	F: 5′‐CATGTACGTTGCTATCCAGGC ‐3′
	R: 5′‐ CTCCTTAATGTCACGCACGAT ‐3′

### 2.6. Western Blot

Differently treated A549 cells were used to prepare protein samples with RIPA buffer supplemented with a 1% protease inhibitor. The protein concentration was determined using a BCA assay kit (P0009, Beyotime, China). Protein samples were separated by sodium dodecyl sulfate‒polyacrylamide gel electrophoresis (SDS‒PAGE), and the separated proteins were transferred to polyvinylidene fluoride (PVDF) membranes (FFP39, Beyotime, China). The membranes were blocked with 5% skim milk at room temperature for 1.5 h and incubated with primary antibodies against HDAC4 (1:1000, 17449‐1‐AP, Proteintech, USA), SLC38A2 (1:2000, 25928‐1‐AP, Proteintech, USA), HIF‐1α (1:1000, A26889, ABclonal, China), Nrf2 (1:1000, ab62352, Abcam, UK), HO‐1 (1:1000, 66743‐1‐Ig, Proteintech, USA), NQO1 (1:5000, 67240‐1‐Ig, Proteintech, USA), and β‐actin (1:1000, ab8227, Abcam, UK) at 4°C overnight. After overnight incubation, the membranes were incubated with secondary antibodies at room temperature for 2 h. The protein bands were visualized using an enhanced chemiluminescence (ECL) kit (WBKLS0500, Millipore, USA). All protein band gray values were normalized to the internal reference protein β‐actin to correct for sample loading differences, and quantitative analysis of relative protein expression levels was performed using ImageJ software.

### 2.7. Flow Cytometry

The expression of the surface markers CD133 and CD44 on A549 cells was detected by flow cytometry. In brief, A549 cells were dissociated with trypsin‐EDTA, washed, and resuspended in phosphate‐buffered saline (PBS). FITC‐labeled anti‐CD133 (10 μL; Cat Number 567033, BD Biosciences, NJ, USA) and anti‐CD44 (10 μL; Cat Number 560977, BD Biosciences, NJ, USA) antibodies were added to 100 μL of the cell suspension. After incubation in the dark at 4°C for 15 min and two PBS washes, the cells were resuspended in 300 μL of PBS, and data were acquired using a flow cytometer.

### 2.8. Glutamine Metabolism Index Detection

Glutamine (ab197011, Abcam, UK), glutamate (ab83389, Abcam, UK), α‐ketoglutarate (ab83431, Abcam, UK), and glutaminase (GLS) activity (ab284547, Abcam, UK) assay kits were used to measure intracellular glutamine uptake, glutamate production, α‐ketoglutarate production, and GLS activity, respectively, in A549 cells.

### 2.9. DCFH‐DA Staining for Cellular ROS Detection

Intracellular ROS levels in A549 cells were detected using a DCFH‐DA fluorescent probe assay (HY‐D0940, MedChem Express, USA). In brief, A549 cells subjected to different treatments were washed with a serum‐free medium to remove residual serum and culture impurities. The cells were subsequently incubated with diluted DCFH‐DA working solution at 37°C for 20 min in the dark. After incubation, the cells were rinsed thoroughly with a serum‐free medium to eliminate unbound probes. The fluorescence intensity of the intracellular ROS was observed and photographed using a fluorescence microscope.

### 2.10. Measurement of the GSH/oxidized glutathione (GSSG) Ratio

The levels of total glutathione (T‐GSH) and GSSG in A549 cells were measured using a GSH/GSSG ratio detection kit (E‐BC‐K097‐M, Elabscience, China) following the manufacturer’s instructions. Briefly, treated A549 cells were lysed and centrifuged to collect the supernatant for subsequent detection. The absorbance of the samples was detected at 412 nm using a microplate reader. The contents of T‐GSH and GSSG were calculated according to the standard curve, and the GSH/GSSG ratio was further analyzed to evaluate the intracellular redox status.

### 2.11. Promoter–Luciferase Reporter Assay

The wild‐type SLC38A2 (WT‐SLC38A2) promoter sequence containing the predicted binding site and the mutant SLC38A2 (MUT‐SLC38A2) sequence with the predicted site mutation were cloned and inserted into a firefly luciferase reporter vector. A549 cells were cotransfected with the recombinant reporter plasmid (WT or MUT), a Renilla luciferase internal control plasmid, and either a HIF‐1α overexpression plasmid or an empty vector plasmid. Forty‐eight hours posttransfection, the cell lysates were collected to measure firefly and Renilla luciferase activities. Relative luciferase activity was calculated by normalizing the firefly luciferase signal to the Renilla luciferase signal.

### 2.12. Chromatin Immunoprecipitation Quantitative Polymerase Chain Reaction (ChIP‐qPCR)

ChIP‐qPCR was performed to detect the enrichment of HIF‐1α on the promoter region of SLC38A2 (SNAT2) in A549 cells. Briefly, treated A549 cells were cross‐linked with formaldehyde to fix protein‒DNA interactions, followed by chromatin fragmentation via ultrasonic shearing. The cell lysates were immunoprecipitated overnight at 4°C with an anti‐HIF‐1α (ab179483, Abcam, UK) antibody, with normal IgG serving as the negative control. After elution and reverse cross‐linking, the enriched DNA fragments were purified. Quantitative PCR was then conducted using specific primers targeting the SLC38A2 promoter region. The relative enrichment level of HIF‐1α binding to the SLC38A2 promoter was calculated and normalized to that of the input control.

### 2.13. Establishment of Animal Models

Ten 5‐week‐old male BALB/c nude mice weighing 16–18 g were obtained from the Animal Experiment Center of Kunming Medical University. All the nude mice were randomly divided into two groups: the sh‐NC group (*n* = 5) and the sh‐HDAC4 group (*n* = 5). The sample size (*n* = 5 per group) was determined based on previous similar studies [26] and our pilot experiments to ensure sufficient statistical power. All animal procedures were approved by the Experimental Animal Ethics Committee of Yunnan Zequn Biotechnology Co., Ltd. (Approval Number ZQSW21‐2501‐12). In the sh‐HDAC4 group, 100 µL of A549 cell suspension with stable knockdown of HDAC4 (sh‐HDAC4) at a cell density of 1 × 10^6^ cells/mL was subcutaneously injected into the right dorsal region of the nude mice. A549 cells stably transfected with the knockdown control (sh‐NC) were injected into the sh‐NC group in the same manner. Tumor growth was measured every 7 days using a Vernier caliper. Repeated‐measures statistical analysis was applied for the longitudinal analysis of tumor growth curves. The tumor volume was calculated using the formula as follows:

volumemm3=0.5× length×width2.

After 28 days of injection, the nude mice were euthanized, the tumors were excised and weighed, and the tumor samples were processed into paraffin‐embedded sections for immunohistochemistry.

### 2.14. Immunohistochemistry

The paraffin‐embedded tumor tissues from the nude mice were subjected to dewaxing, rehydration, and antigen retrieval. The slides were subsequently incubated with primary antibodies against HIF‐1α (1:200, 48085, Cell Signaling Technology, USA), SLC38A2 (1:200, 25928‐1‐AP, Proteintech, USA), SOX2 (1:100, ab97959, Abcam, UK), and octamer‐binding transcription factor 4 (OCT4) (1:200, PA1‐16943, Thermo Fisher Scientific, USA) at 4°C overnight. After overnight incubation, secondary antibodies were applied, followed by DAB color development and observation under an optical microscope.

### 2.15. Bioinformatics Analysis

To clarify the roles of HDAC4 in regulating cancer stemness and metabolism in NSCLC, transcriptomic data from TCGA and GEO were integrated for correlation analysis in this study. RNA‐seq expression matrices for TCGA lung adenocarcinoma (TCGA‐LUAD) and TCGA lung squamous cell carcinoma (TCGA‐LUSC) were downloaded from the University of California, Santa Cruz Xena (UCSC Xena) platform. Tumor samples were screened according to TCGA sample barcodes, and samples annotated with the sample type code “01” were defined as tumor tissues. The expression levels of HDAC4 and markers associated with cancer stemness and metabolism, including SLC38A2, SOX2, NANOG, CD44, SLC7A5, and ALDH1A1, were determined. Spearman’s rank correlation coefficients between HDAC4 and each marker were calculated for the LUAD and LUSC cohorts. Three external validation datasets from GEO, namely, GSE18842, GSE19188, and GSE19804, were included in the analysis. The expression values of HDAC4, SLC38A2, NANOG, CD44, and ALDH1A1 were extracted based on platform annotation files. For genes matched with multiple probes, the probe with the greatest difference in expression was selected as the representative probe. All correlation analyses were performed using Spearman’s rank correlation test, and the Benjamini–Hochberg method was applied for the false discovery rate (FDR) correction of *p*‐values. Correlation results were visualized via heatmaps. Furthermore, to intuitively illustrate the correlations between HDAC4 and core mechanistic markers, SLC38A2, SOX2, and SLC7A5 were selected from TCGA‐LUAD cohort, while SOX2, SLC7A5, and ALDH1A1 were chosen from TCGA‐LUSC cohort for scatter plot correlation analysis with fitted trend lines.

### 2.16. Statistical Analysis

The experimental data were analyzed using GraphPad Prism 8 software (GraphPad, USA). Quantitative results are presented as the mean ± standard deviation (SD). Independent *t*‐tests were used for comparisons between two groups, whereas one‐way or two‐way analysis of variance (ANOVA) was used for multiple‐group comparisons, followed by Tukey’s post‐hoc multiple comparison test to identify significant differences between individual groups. The cell experiments were repeated three times, and the animal experiments were repeated five times with independent biological replicates. A *p*‐value less than 0.05 was defined as statistically significant.

## 3. Results

### 3.1. Correlations of HDAC4 Expression With Cancer Stemness and Metabolic Markers in NSCLC

To explore the role of HDAC4 in the regulation of cancer stemness and metabolism in NSCLC, correlation analysis was performed based on data from TCGA and GEO databases. TCGA correlation heatmaps revealed that in TCGA‐LUAD and TCGA‐LUSC tumor samples, HDAC4 expression levels were significantly positively correlated with the expression of multiple cancer stemness‐related genes (SOX2, NANOG, CD44, and ALDH1A1) and amino acid metabolism‐related transporters (SLC38A2 and SLC7A5) (Figure 1A), suggesting that HDAC4 may be associated with cancer stemness and amino acid metabolism in NSCLC. GEO correlation heatmaps further verified that HDAC4 expression was significantly positively correlated with the expression of the amino acid metabolism‐related transporter SLC38A2 in the GSE19804 cohort and was significantly positively correlated with the expression of the cancer stemness markers NANOG, CD44, and ALDH1A1 in the GSE19188 cohort (Figure 1B). The GEO datasets effectively corroborated the involvement of HDAC4 in the regulation of cancer stemness and amino acid metabolism in NSCLC. Finally, scatter fitting analysis revealed that HDAC4 expression was significantly positively correlated with SLC38A2, SOX2, and SLC7A5 expression in TCGA‐LUAD cohort (Figure 1C), whereas HDAC4 expression was significantly positively correlated with SOX2, SLC7A5, and ALDH1A1 expression in TCGA‐LUSC cohort (Figure 1D). These findings support the potential association of HDAC4 with the cancer stemness status and amino acid metabolism in NSCLC.

**Figure 1:**
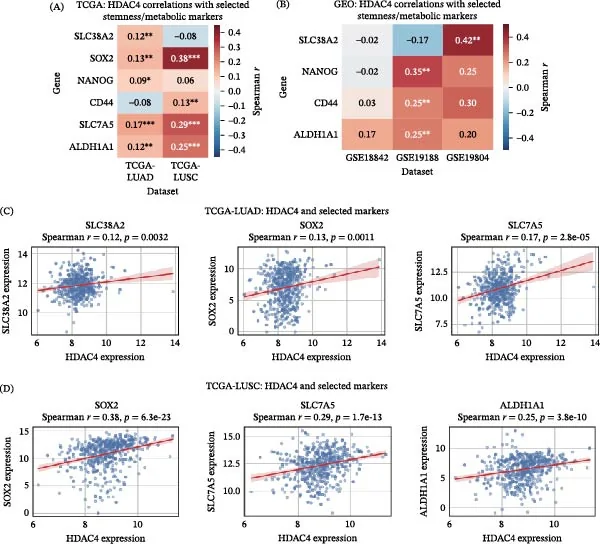
Correlations of HDAC4 expression with cancer stemness and metabolic markers in NSCLC. (A) Heatmap showing the correlation between HDAC4 and cancer stemness/metabolic markers in TCGA‐LUAD and TCGA‐LUSC cohorts. (B) External validation of the correlation between HDAC4 and key markers in multiple GEO NSCLC datasets (GSE18842, GSE19188, and GSE19804). (C) Scatter plots with fitted trend lines showing the correlations of HDAC4 with SLC38A2, SOX2, and SLC7A5 in TCGA‐LUAD cohort. (D) Scatter plots with fitted trend lines showing the correlations of HDAC4 with SOX2, SLC7A5, and ALDH1A1 in TCGA‐LUSC cohort. HDAC4, histone deacetylase 4; NSCLC, nonsmall cell lung cancer; TCGA, The Cancer Genome Atlas; LUAD, lung adenocarcinoma; LUSC, lung squamous cell carcinoma; GEO, Gene Expression Omnibus; SLC38A2, solute carrier family 38 member 2; SOX2, SRY‐box transcription factor 2; SLC7A5, solute carrier family 7 member 5; ALDH1A1, aldehyde dehydrogenase 1 family member A1.

### 3.2. Knockdown of HDAC4 Inhibits the Stemness of A549 Cells

Based on the abovementioned correlation between HDAC4 expression and cancer stemness markers in NSCLC, this study further investigated the functional role of HDAC4 in regulating cancer stemness. Stable HDAC4 knockdown was achieved in A549 cells, with the sh‐HDAC4#1 and sh‐HDAC4#2 groups both showing significantly reduced HDAC4 expression, and the sh‐HDAC4#2 group exhibiting a more pronounced decrease (Figure 2A,B). Therefore, sh‐HDAC4#2 was selected for subsequent experiments. Notably, CCK‐8 assays confirmed that HDAC4 knockdown did not affect A549 cell viability (Figure 2C), excluding the potential confounding effect of reduced proliferation on subsequent functional readouts. We then found that HDAC4 knockdown significantly suppressed the sphere‐forming ability of A549 cells (Figure 2D). The RT‐qPCR results revealed that compared with the sh‐NC group, the HDAC4 knockdown group had significantly lower mRNA expression levels of the stemness‐related markers SOX2, OCT4, and NANOG in A549 cells (Figure 2E). Flow cytometry analysis revealed that HDAC4 knockdown significantly decreased the proportion of CD133‐ or CD44‐positive cells (Figure 2F). In addition, HDAC4 knockdown suppressed the migration (Figure 2G) and invasion (Figure 2H) abilities of A549 cells. In summary, these results demonstrate that HDAC4 knockdown suppresses the stemness in A549 cells.

Figure 2Knockdown of HDAC4 inhibits the stemness of A549 cells. (A) RT‐qPCR detection of HDAC4 transduction efficiency. (B) Western blot detection of HDAC4 transduction efficiency. (C) CCK‐8 assay for A549 cell viability. (D) Sphere formation assay to assess the spheroid‐forming ability of A549 cells; scale bar: 100 μm. (E) RT‐qPCR detection of the expression of stemness‐related markers SOX2, OCT4, and NANOG mRNA in A549 cells. (F) Flow cytometry analysis of the proportions of CD133‐ and CD44‐positive A549 cells. (G) Transwell assay to evaluate the migratory ability of A549 cells; scale bar: 100 μm. (H) Transwell assay to evaluate the invasion ability of A549 cells; scale bar: 100 μm.  ^∗∗^ *p* < 0.01,  ^∗∗∗^ *p* < 0.001. Data are presented as mean ± SD. All experiments were performed with three independent biological replicates. Statistical analysis was conducted using one‐way ANOVA for panels (A) and (B) and Student’s *t*‐test for panels (C)–(H). RT‐qPCR, reverse transcription quantitative polymerase chain reaction; CCK‐8, Cell Counting Kit 8; SD, standard deviation; ANOVA, analysis of variance; OCT4, octamer‐binding transcription factor 4; NANOG, nanog homeobox; sh‐NC: short hairpin RNA negative control; sh‐HDAC4: short hairpin RNA targeting HDAC4; FITC: fluorescein isothiocyanate. The original, uncropped western blot images for Figure S2B are provided in the Supporting Information.

**Figure:**
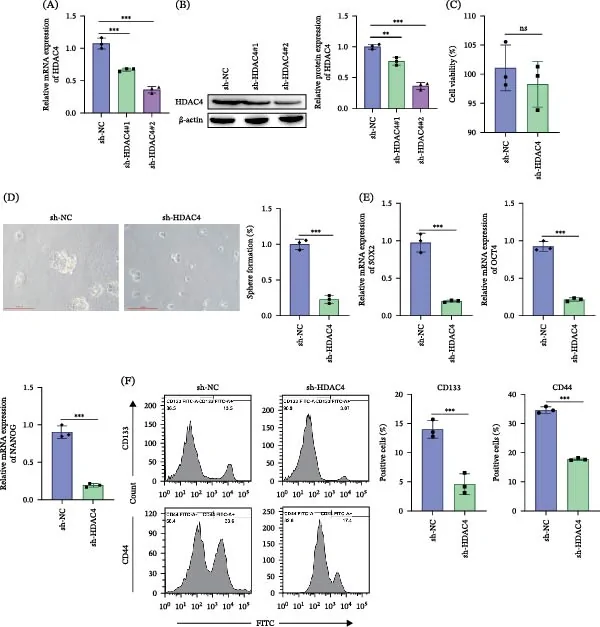

**Figure:**
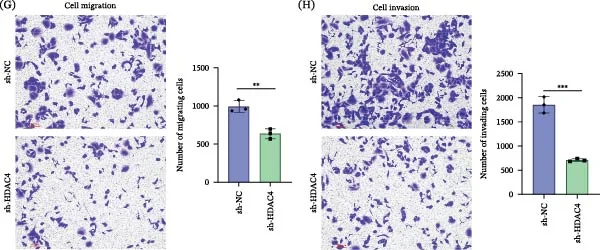

### 3.3. Knockdown of HDAC4 Inhibits Glutamine Metabolism in A549 Cells

Studies have shown that glutamine metabolism is closely associated with cancer stemness progression [27]. Given that the results of the aforementioned bioinformatics analysis suggested an association between HDAC4 and the amino acid metabolism status in NSCLC, the present study further investigated the regulatory role of HDAC4 in glutamine metabolism in NSCLC cells. The results of functional experiments revealed that HDAC4 knockdown significantly inhibited glutamine uptake, glutamate production, and α‐ketoglutarate production in A549 cells (Figure 3A–C) and simultaneously significantly reduced GLS activity in A549 cells (Figure 3D). Furthermore, we found that HDAC4 knockdown was accompanied by increased intracellular ROS levels, a decreased GSH/GSSG ratio, and decreased protein expression levels of Nrf2, HO‐1, and NQO1 (Figure 3E–G), further supporting the redox imbalance resulting from impaired glutamine metabolism. Collectively, these results indicate that HDAC4 knockdown inhibits glutamine metabolism in A549 cells.

**Figure 3:**
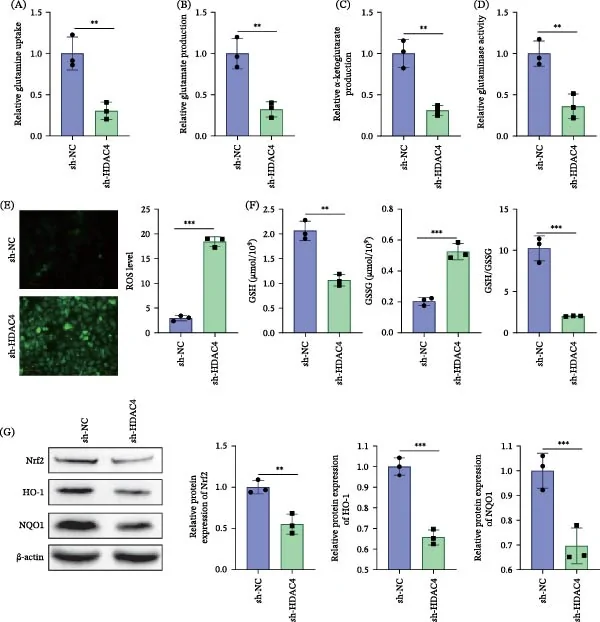
Knocking down HDAC4 inhibits glutamine metabolism in A549 cells. (A) Glutamine uptake levels in A549 cells. (B) Glutamate production levels in A549 cells. (C) α‐Ketoglutarate production levels in A549 cells. (D) Measurement of GLS activity in A549 cells. (E) DCFH‐DA staining was used to measure the level of ROS in cells. (F) A kit was used to measure the GSH/GSSG ratio. (G) Western blotting was used to detect the expression of Nrf2, HO‐1, and NQO1.  ^∗∗^ *p* < 0.01,  ^∗∗∗^ *p* < 0.001. Data are presented as mean ± SD. All experiments were performed with three independent biological replicates. Statistical analysis was conducted using Student’s *t*‐test for panels (A)‒(G). GLS, glutaminase; DCFH‐DA, 2^′^,7^′^ dichlorodihydrofluorescein diacetate; ROS, reactive oxygen species; GSH, reduced glutathione; GSSG, oxidized glutathione; Nrf2, nuclear factor erythroid 2 related factor 2; HO‐1, heme oxygenase 1; NQO1: NAD(P)H quinone dehydrogenase 1. The original, uncropped western blot images for Figure S3G are provided in the Supporting Information.

### 3.4. Inhibition of Glutamine Metabolism Attenuates the Enhancing Effect of HDAC4 Overexpression on Stemness in A549 Cells

Next, to investigate the impact of HDAC4‐regulated glutamine metabolism on the stemness of A549 cells, we overexpressed HDAC4 in A549 cells. Analysis of the HDAC4 overexpression efficiency revealed that HDAC4 expression was significantly upregulated in the OE‐HDAC4 group compared with that in the OE‐NC group, indicating successful overexpression (Figure 4A,B). A sphere formation assay revealed that HDAC4 overexpression enhanced the sphere‐forming ability of A549 cells, whereas treatment with the glutamine metabolism inhibitor CB‐839 significantly weakened this ability and partially reversed the effect of HDAC4 overexpression (Figure 4C). RT‐qPCR results showed that HDAC4 overexpression significantly promoted the mRNA expression of the stemness‐related markers SOX2, OCT4, and NANOG in A549 cells, whereas CB‐839 treatment reversed the changes in the expression of these indicators (Figure 4D). Flow cytometry analysis revealed that HDAC4 overexpression significantly increased the proportions of CD133‐ and CD44‐positive cells, whereas CB‐839 treatment reversed these changes (Figure 4E). Additionally, CB‐839 treatment reversed the promoting effect of HDAC4 overexpression on A549 cell migration and invasion (Figure 4F,G). These results indicate that HDAC4 enhances the stemness of A549 cells by promoting glutamine metabolism.

Figure 4Inhibition of glutamine metabolism attenuates the enhancing effect of HDAC4 overexpression on stemness in A549 cells. (A) RT‐qPCR detection of HDAC4 transduction efficiency. (B) Western blot detection of HDAC4 transduction efficiency. (C) Sphere formation assay to assess the spheroid‐forming ability of A549 cells; scale bar: 100 μm. (D) RT‐qPCR detection of the expression of the stemness‐related markers SOX2, OCT4, and NANOG mRNA in A549 cells. (E) Flow cytometry analysis of the proportions of CD133‐ and CD44‐positive cells in A549 cells. (F) Transwell assay to evaluate the migration ability of A549 cells; scale bar: 100 μm. (G) Transwell assay to evaluate the invasion ability of A549 cells; scale bar: 100 μm.  ^∗^ *p* < 0.05,  ^∗∗^ *p* < 0.01,  ^∗∗∗^ *p* < 0.001. Data are presented as mean ± SD. All experiments were performed with three independent biological replicates. Statistical analysis was conducted using Student’s *t*‐test for panels (A) and (B) and one‐way ANOVA for panels (C)‒(G). OE‐NC, overexpression negative control; OE‐HDAC4, overexpression HDAC4. The original, uncropped Western blot images for Figure S4B are provided in the Supporting Information.

**Figure:**
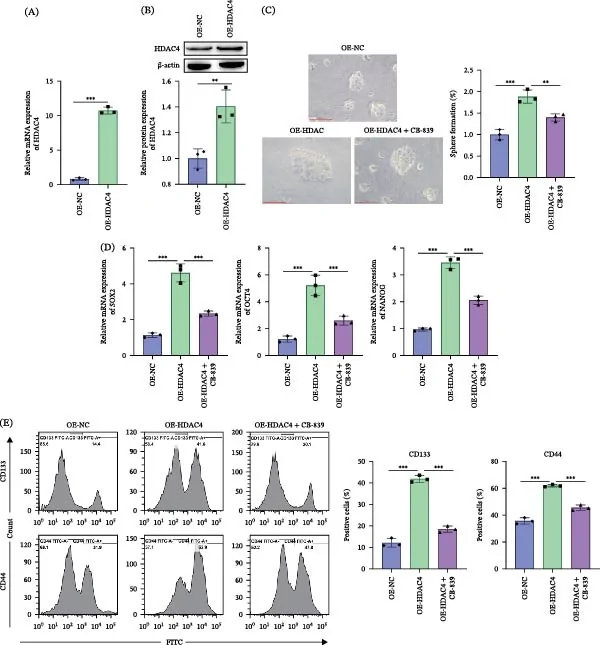

**Figure:**
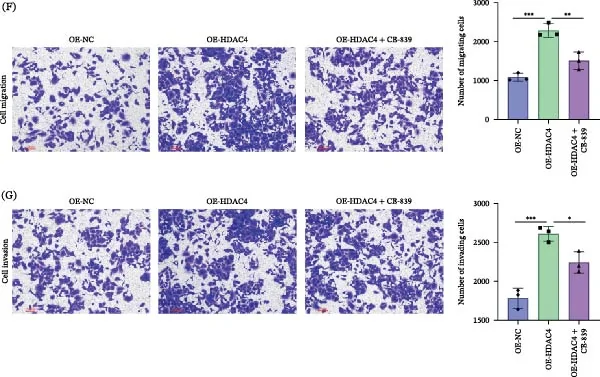

### 3.5. HDAC4 Promotes SLC38A2 Expression Through HIF‐1α

Previous studies have shown that the transporter protein SLC38A2 can activate glutamine metabolism [28]. Additionally, research has indicated that HDAC4 can stabilize the expression of HIF‐1α, which in turn promotes the transcription of SLC38A2 [25, 29]. Accordingly, in this study, the regulatory effects of HDAC4 on the expression of HIF‐1α and SLC38A2 in A549 cells were investigated. Western blot results revealed that the protein levels of both SLC38A2 and HIF‐1α were significantly lower in the sh‐HDAC4 group than in the sh‐NC group but markedly greater in the OE‐HDAC4 group than in the OE‐NC group (Figure 5A–D). To further verify the direct transcriptional regulation of SLC38A2 by HIF‐1α, JASPAR database prediction was first performed, and the results revealed potential binding sites of HIF‐1α in the promoter region of SLC38A2 (Figure 5E). ChIP‐qPCR further confirmed the significant enrichment of HIF‐1α at the SLC38A2 promoter (Figure 5F). The results of the dual‐luciferase reporter assay demonstrated that HIF‐1α overexpression significantly increased the luciferase activity driven by the SLC38A2 promoter (Figure 5G). Collectively, these results indicate that HIF‐1α directly binds to and activates the transcription of SLC38A2. Furthermore, to investigate whether HDAC4 regulates SLC38A2 expression through HIF‐1α in A549 cells, we overexpressed HIF‐1α in A549 cells (Figure 5H,I). A rescue experiment revealed that HIF‐1α overexpression effectively reversed the inhibitory effect of HDAC4 knockdown on the SLC38A2 expression in A549 cells (Figure 5J). To further consolidate the molecular evidence that HDAC4 regulates SLC38A2 in a HIF‐1α‐dependent manner, we established a HIF‐1α knockdown cell model for reverse rescue experiments. RT‐qPCR and Western blot results confirmed that sh‐HIF‐1α significantly decreased HIF‐1α mRNA and protein expression levels in A549 cells (Figure 5K,L). The results of a subsequent rescue experiment showed that HDAC4 overexpression alone significantly upregulated SLC38A2 protein expression, whereas combined knockdown of HIF‐1α effectively reversed the HDAC4 overexpression‐mediated upregulation of SLC38A2 (Figure 5M). These results indicate that HDAC4 promotes the SLC38A2 expression via HIF‐1α.

**Figure 5:**
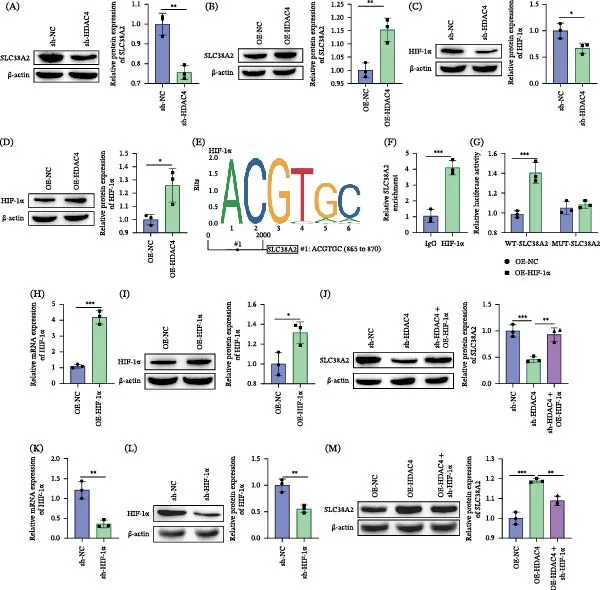
HDAC4 promotes SLC38A2 expression through HIF‐1α. (A) Western blot detection of SLC38A2 expression in A549 cells with HDAC4 knockdown. (B) Western blot detection of SLC38A2 expression in A549 cells with HDAC4 overexpression. (C) Western blot detection of HIF‐1α expression in A549 cells with HDAC4 knockdown. (D) Western blot detection of HIF‐1α expression in A549 cells with HDAC4 overexpression. (E) JASPAR analysis of the binding sites of HIF‐1α and the promoter of SLC38A2. (F) ChIP‐qPCR detection of the enrichment level of HIF‐1α at the SLC38A2 promoter in A549 cells. (G) A promoter‐luciferase reporter assay was used to verify the binding of HIF‐1α to the SLC38A2 promoter. (H) RT‐qPCR detection of HIF‐1α mRNA expression in A549 cells with HIF‐1α overexpression. (I) Western blot detection of HIF‐1α expression in A549 cells with HIF‐1α overexpression. (J) Western blot detection of SLC38A2 expression in A549 cells. (K) RT‐qPCR detection of HIF‐1α mRNA expression in A549 cells with HIF‐1α knockdown. (L) Western blot detection of HIF‐1α expression in A549 cells with HIF‐1α knockdown. (M) Western blot detection of SLC38A2 expression in A549 cells.  ^∗^ *p* < 0.05,  ^∗∗^ *p* < 0.01,  ^∗∗∗^ *p* < 0.001. Data are presented as mean ± SD. All experiments were performed with three independent biological replicates. Statistical analysis was conducted using Student’s *t*‐test for panels (A)–(D), (F)–(I) and (K)–(L) and one‐way ANOVA for panels (J) and (M). HIF‐1α, hypoxia‐inducible factor‐1 subunit alpha; ChIP‐qPCR, chromatin immunoprecipitation quantitative polymerase chain reaction; IgG, immunoglobulin G; OE‐HIF‐1α, overexpression HIF‐1α; sh‐HIF‐1α, short hairpin RNA targeting HIF‐1α. The original, uncropped Western blot images for Figure S5A–D, I,J, L,M are provided in the Supporting Information.

### 3.6. Knockdown of HDAC4 Inhibits Tumor Growth and Stemness In Vivo

Finally, to further evaluate the impact of HDAC4 on tumor growth and stemness in vivo, we subcutaneously injected A549 cells with stable knockdown of HDAC4 (sh‐HDAC4) and the control (sh‐NC) into BALB/c nude mice to establish a tumor‐bearing model and detected relevant indicators. The results showed that the knockdown of HDAC4 inhibited tumor growth (Figure 6A–C). Immunohistochemical staining revealed that compared with those in the sh‐NC group, the expression levels of HIF‐1α, SLC38A2, SOX2, and OCT4 in tumor tissues in the sh‐HDAC4 group were significantly lower (Figure 6D). These results indicate that knockdown of HDAC4 downregulates HIF‐1α and SLC38A2 expression, thereby inhibiting tumor growth and stemness in vivo.

**Figure 6:**
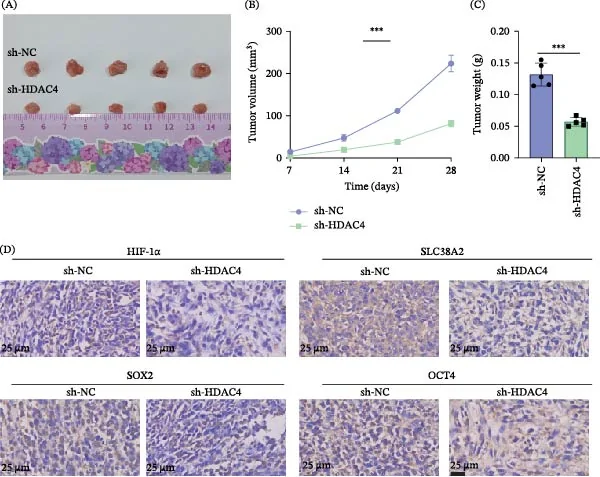
Knockdown of HDAC4 inhibits tumor growth and stemness in vivo. (A) Tumor morphology in nude mice. (B) Tumor volume in nude mice. (C) Tumor weight in nude mice. (D) Immunohistochemical detection of HIF‐1α, SLC38A2, SOX2, and OCT4 expression in tumor tissues; scale bar: 25 μm.  ^∗∗∗^ *p* < 0.001. Data are presented as mean ± SD. All experiments were performed with five independent biological replicates. Statistical analysis was conducted using two‐way ANOVA for panel (B) and Student’s *t*‐test for panel (C).

## 4. Discussion

NSCLC is a common malignant tumor that poses serious threats to human health and life. Studies have shown that cancer stemness plays a significant role in the progression of malignant tumors, including metastasis and low sensitivity to antitumor therapies [30]. Therefore, exploring new interventions targeting cancer stemness holds promise for improving NSCLC progression. In this study, we found that knocking down the HDAC4 expression inhibited the stemness of A549 cells. Mechanistically, HDAC4 enhances glutamine metabolism by upregulating HIF‐1α to promote SLC38A2 expression, thereby promoting NSCLC stemness.

HDAC4 is a histone deacetylase that plays crucial roles in regulating cell growth, survival, proliferation, and differentiation [31]. Research has elucidated the role played by HDAC4 in lung cancer. A study by Jin et al. [32] reported that miR‐520b inhibits lung cancer cell growth by targeting HDAC4. Shao et al. [33] demonstrated that the senescence marker protein 30 suppresses tumor growth by reducing HDAC4 expression in NSCLC. In addition, studies have shown that HDAC4 can regulate cancer stemness and participate in tumor progression. For example, Xu et al. [20] found that HDAC4‐mediated deacetylation of GLS promotes glioma cancer stemness. However, whether HDAC4 affects NSCLC stemness remains unclear. In this study, we found that knocking down HDAC4 expression significantly inhibited the spheroid formation, cell migration, and invasion abilities of A549 cells. Furthermore, HDAC4 knockdown suppressed the expression of the stemness‐related markers SOX2, OCT4, and NANOG in A549 cells and reduced the proportions of CD133‐ and CD44‐positive cells. Additionally, HDAC4 knockdown similarly inhibited tumor growth in nude mice and suppressed the expression of SOX2 and OCT4. Our findings are the first to demonstrate that HDAC4 inhibits NSCLC stemness.

Glutamine can meet the energy demands of tumor cells, and glutamine metabolism in tumors mediates various biosynthetic processes, including energy production, redox regulation, gene transcription, and intracellular signal transduction [34, 35]. Glutamine metabolism plays a significant role in the progression of NSCLC. Research by Qu et al. [36] found that MYLK‐AS1 can increase NSCLC resistance to EGFR inhibitors by augmenting glutamine metabolism. Xia et al. [37] demonstrated that targeted inhibition of glutamine metabolism can increase the antitumor efficacy of selumetinib in KRAS‐mutant NSCLC. Similarly, Cai et al. [38] revealed that Circ_0000808 regulates glutamine metabolism through the miR‐1827/SLC1A5 axis, thereby promoting NSCLC development. Interestingly, previous studies have indicated that glutamine metabolism is associated with the cancer stemness progression. For instance, Li et al. [39] found that inhibiting glutamine metabolism suppresses pancreatic cancer stem cell characteristics and sensitizes cells to radiotherapy. In this study, we discovered that knocking down HDAC4 inhibited glutamine uptake, glutamate production, and α‐ketoglutarate generation and reduced GLS activity in A549 cells. Further investigations revealed that treatment with the glutamine metabolism inhibitor CB‐839 reversed the promoting effects of HDAC4 overexpression on NSCLC stemness and malignant proliferation. Our study supports that HDAC4 promotes NSCLC stemness by enhancing glutamine metabolism.

SLC38A2 is one of the key regulators of glutamine metabolism. Wu et al. [13] demonstrated that the interaction between HHLA2 and KIR3DL3 inhibits glutamine metabolism in CD8^+^ T cells by suppressing key glutamine transporters and metabolic enzymes, including SLC1A5, SLC38A2, and ADHFE1, in an ERK/MAPK‐dependent manner, thereby promoting immune evasion in EGFR‐mutant lung cancer. In this study, we revealed that knocking down HDAC4 expression suppressed the SLC38A2 expression in A549 cells, whereas overexpressing HDAC4 increased the SLC38A2 expression. In vivo studies revealed that HDAC4 knockdown inhibited the SLC38A2 expression. Our results indicate that HDAC4 promotes glutamine metabolism by upregulating the SLC38A2 expression, thereby enhancing cancer stemness in NSCLC.

HIF‐1α is a hypoxia‐inducible factor involved in regulating various physiological processes and is closely associated with the progression of NSCLC. A study by Hua et al. [40] demonstrated that hypoxia‐induced lncRNA‐AC020978 expression promotes proliferation and glycolytic metabolism in NSCLC by regulating the PKM2/HIF‐1α axis. Similarly, research by Zhao et al. [41] reported that EZH2 regulates the expression of the immunosuppressive molecule PD‐L1 through HIF‐1α, thereby delaying NSCLC progression by enhancing antitumor immune responses. Additionally, the study by Li et al. [42] revealed that hypoxia‐induced HIF‐1α expression can drive NSCLC cell stemness and cisplatin resistance by upregulating Tie1 expression. Notably, studies have indicated that the accumulation of HDAC4 in the nucleus leads to reduced acetylation of HIF‐1α, thereby increasing HIF‐1α stability and transcriptional activity, which strengthens cellular adaptive responses to hypoxia [29]. Consistent with previous findings, in this study, we observed that knocking down HDAC4 expression inhibited HIF‐1α expression in A549 cells, whereas overexpressing HDAC4 increased the HIF‐1α expression. Furthermore, research by Morotti et al. [25] reported that HIF‐1α upregulates SLC38A2, leading to complete resistance to antiestrogen and partial anti‐VEGF therapies in breast cancer. Consistent with the findings of previous studies, we discovered that knocking down HDAC4 expression suppressed HIF‐1α expression in nude mouse xenograft tissues and that in vitro overexpression of HIF‐1α reversed the inhibitory effect of HDAC4 knockdown on SLC38A2 expression. These results indicate that HDAC4 promotes SLC38A2 expression by upregulating the HIF‐1α expression.

In summary, this study is the first to comprehensively demonstrate that HDAC4 can upregulate HIF‐1α to transcriptionally activate SLC38A2‐mediated glutamine metabolism, thereby regulating cancer stemness phenotypes and to establish a novel HDAC4–HIF‐1α–SLC38A2 epigenetic–metabolism–stemness regulatory axis, enhancing the multidimensional regulatory network underlying NSCLC malignant progression. However, as all in vitro experiments in this study were conducted exclusively in A549 cells, certain limitations exist due to the heterogeneity of NSCLC. The generalizability of these findings was further validated using additional NSCLC cell lines. Overall, the results of this study provide a novel potential target and a theoretical basis for targeted intervention and mechanistic research in NSCLC.

## Author Contributions

All the authors contributed substantially to this manuscript. Conceptualization, project administration, writing – original draft: Changxian Chen and Xiaoming Jiang. Data curation, software: Liju Zhang and Mingyu Xu. Formal analysis, methodology: Zhenwu Yao and Jiaxue Sun. Funding acquisition, resources: Weijun Liu. Investigation, supervision: Weimin Bao. Validation: Changxian Chen and Weimin Bao. Visualization: Xiaoming Jiang and Weijun Liu. Writing – review and editing: Weimin Bao and Weijun Liu.

## Funding

This study was supported by the Joint Special Fund of Kunming Medical University‐Key Project (Grant 202301AY070001‐016).

## Disclosure

All authors read and approved the final manuscript.

## Ethics Statement

All animal experimental protocols were approved by the Experimental Animal Ethics Committee of Yunnan Zequn Biotechnology Co., Ltd. (Approval Number ZQSW21‐2501‐12). All procedures were carried out in accordance with the ARRIVE guidelines 2.0, the U.K. Animals (Scientific Procedures) Act, 1986 and associated guidelines, and the National Institutes of Health guide for the care and use of laboratory animals (NIH Publications Number 8023, revised 1978).

## Conflicts of Interest

The authors declare no conflicts of interest.

## Supporting Information

Additional supporting information can be found online in the Supporting Information section.

## Supporting information


**Supporting Information** The original, uncropped western blot images for Figures S2B, S3G, S4B, and S5A–D, I,J, L,M are provided in the Supporting Information.

## Data Availability

Data are available upon request from the authors.
