# Supplementary material for: HDAC4 Promotes Cancer Stemness in Nonsmall Cell Lung Cancer by Upregulating SLC38A2 Expression via HIF‐1α to Enhance Glutamine Metabolism
Source: Anal Cell Pathol (Amst). 2026 Sep 28;2026:2525721. doi: 10.1155/ancp/2525721 (PMC13617536; doi:10.1155/ancp/2525721)
Supplement: Supplementary file 1 [file ANCP-2026-2525721-s001.docx]

Figure2B

HDAC4(140 kDa)

Repeat 1	Repeat 2	Repeat 3

β-actin (42 kDa)

Repeat 1	Repeat 2	Repeat 3

Figure3G

Nrf2 (110 kDa)

Repeat 1	Repeat 2	Repeat 3

HO-1 (33 kDa)

Repeat 1	Repeat 2	Repeat 3

NQO1 (33 kDa)

Repeat 1	Repeat 2	Repeat 3

β-actin (42 kDa)

Repeat 1	Repeat 2	Repeat 3

Figure4B

HDAC4(140 kDa)

Repeat 1	Repeat 2	Repeat 3

β-actin (42 kDa)

Repeat 1	Repeat 2	Repeat 3

Figure5A

SLC38A2 (56 kDa)

Repeat 1	Repeat 2	Repeat 3

β-actin (42 kDa)

Repeat 1	Repeat 2	Repeat 3

Figure5B

SLC38A2 (56 kDa)

Repeat 1	Repeat 2	Repeat 3

β-actin (42 kDa)

Repeat 1	Repeat 2	Repeat 3

Figure5C

HIF-1α (120 kDa)

Repeat 1	Repeat 2	Repeat 3

β-actin (42 kDa)

Repeat 1	Repeat 2	Repeat 3

Figure5D

HIF-1α (120 kDa)

Repeat 1	Repeat 2	Repeat 3

β-actin (42 kDa)

Repeat 1	Repeat 2	Repeat 3

Figure5I

HIF-1α (120 kDa)

Repeat 1	Repeat 2	Repeat 3

β-actin (42 kDa)

Repeat 1	Repeat 2	Repeat 3

Figure5J

SLC38A2 (56 kDa)

Repeat 1	Repeat 2	Repeat 3

β-actin (42 kDa)

Repeat 1	Repeat 2	Repeat 3

Figure5L

HIF-1α (120 kDa)

Repeat 1	Repeat 2	Repeat 3

β-actin (42 kDa)

Repeat 1	Repeat 2	Repeat 3

Figure5M

SLC38A2 (56 kDa)

Repeat 1	Repeat 2	Repeat 3

β-actin (42 kDa)

Repeat 1	Repeat 2	Repeat 3
